# Cardiac Dysregulation and Myocardial Injury in a 6-Hydroxydopamine-Induced Rat Model of Sympathetic Denervation

**DOI:** 10.1371/journal.pone.0133971

**Published:** 2015-07-31

**Authors:** Yue-Hua Jiang, Ping Jiang, Jin-long Yang, Du-Fang Ma, Hai-Qing Lin, Wen-ge Su, Zhen Wang, Xiao Li

**Affiliations:** 1 Affiliated Hospital of Shandong University of Traditional Chinese Medicine, Jinan, Shandong, P.R. China; 2 Shandong University of Traditional Chinese Medicine, Jinan, Shandong, P.R. China; University of Buenos Aires, Faculty of Medicine. Cardiovascular Pathophysiology Institute., ARGENTINA

## Abstract

**Background:**

Cardiac sympathetic denervation is found in various cardiac pathologies; however, its relationship with myocardial injury has not been thoroughly investigated.

**Methods:**

Twenty-four rats were assigned to the normal control group (NC), sympathectomy control group (SC), and a sympathectomy plus mecobalamin group (SM). Sympathectomy was induced by injection of 6-OHDA, after which, the destruction and distribution of sympathetic and vagal nerve in the left ventricle (LV) myocardial tissue were determined by immunofluorescence and ELISA. Heart rate variability (HRV), ECG and echocardiography, and assays for myocardial enzymes in serum before and after sympathectomy were examined. Morphologic changes in the LV by HE staining and transmission electron microscope were used to estimate levels of myocardial injury and concentrations of inflammatory cytokines were used to reflect the inflammatory reaction.

**Results:**

Injection of 6-OHDA decreased NE (933.1 ± 179 ng/L for SC *vs*. 3418.1± 443.6 ng/L for NC, *P* < 0.01) and increased NGF (479.4± 56.5 ng/mL for SC *vs*. 315.85 ± 28.6 ng/mL for NC, *P* < 0.01) concentrations. TH expression was reduced, while ChAT expression showed no change. Sympathectomy caused decreased HRV and abnormal ECG and echocardiography results, and histopathologic examinations showed myocardial injury and increased collagen deposition as well as inflammatory cell infiltration in the cardiac tissue of rats in the SC and SM groups. However, all pathologic changes in the SM group were less severe compared to those in the SC group.

**Conclusions:**

Chemical sympathectomy with administration of 6-OHDA caused dysregulation of the cardiac autonomic nervous system and myocardial injuries. Mecobalamin alleviated inflammatory and myocardial damage by protecting myocardial sympathetic nerves.

## Introduction

During the past three decades, the sympathetic nervous system has received increased attention due to its crucial role in cardiovascular medicine. Sympathetic nerve injury, regeneration or remodeling can either accompany or subsequent to myocardial ischemia, necrosis, and remodeling. Excessive sprouting of sympathetic nerves and subsequent hyperinnervation received increased attention due to their associations with fatal arrhythmia and sudden death[1]. Additionally, previous studies have shown that chronic activation of the sympathetic nervous system is a key contributor to inflammatory reactions, cardiac hypertrophy and fibrosis, which result from prior heart disorders [2]. Sympathectomy and β-blockers are widely used to diminish the adverse effects of sympathetic hyperinnervation; however, studies have revealed that cardiac denervation often exists in patients with advanced diabetes, heart failure [3, 4]or a healed myocardial infarction [5]. The effects of cardiac sympathetic denervation on cardiac structure and function have not been adequately studied. It was previously reported that chronic sympathectomy by administration of 6-hydroxydopamine (6-OHDA) accentuated the adverse effects of coronary artery ligation in conscious rats, while normal activity of the cardiac sympathetic system following ligation was not detrimental, and might aid in survival[6]. It is believed that cardiac sympathetic integrity and activity can be impaired by alterations which occur in the nerve terminals and post synaptic β1-AR-AC coupling system during the pathogenesis of diabetes[7].Left ventricular torsion associated with the sympathetic nerve disorder occurs in patients with type 1 diabetes, even when they are not accompanied by coronary heart disease and heart failure [8]. In dog model, cardiac denervation not only failed to cause collateral development but actually had adverse effects that led to an increase in infarct size[9]. Thus, we speculate that cardiac sympathetic denervation is responsible for abnormal changes in cardiac structure and function.

The present study was conducted to test the hypothesis that chemical sympathectomy causes dysregulation of the cardiac autonomic nervous system and myocardial tissue injury. Sympathectomy was achieved by 6-OHDA, which extensively used to chemically induce sympathetic terminal destruction, and to exam the role of the autonomic nervous system in the regulation of cardiovascular functions in experimental animals [10–14]. Besides, study demonstrated that 6-OHDA produces selective degeneration of adrenergic nerve terminals and blockades or destroys adrenergic receptor sites [10] which consisted with the pathogenesis of diabetes [7]. Mecobalamin, the activated form of vitamin B12, displays a special affinity for nerve tissue, where it promotes myelination and transport of the axonal cytoskeleton [15]. Mecobalamin is prescribed to ameliorate various neuropathies, and when administered at continuous high doses, was shown to improve nerve regeneration and function in a rat model of sciatic nerve injury [16]. In Asia, mecobalamin is extensively used in treatment of diabetic peripheral neuropathy, and animal studies have demonstrated its ameliorative effects on peripheral nerve lesions in experimental models of diabetic neuropathy [17]. In the present study, mecobalamin was used as a neuroprotective agent, to offset neurotoxicity of 6-OHDA, allowing us to observe the association between different degrees of sympathetic injury and myocardial injury and exclude the possible myocardial toxicity of 6-OHDA.

## Methods

### Animals and ethics statement

Twenty-four male Sprague- Dawley rats (aged 8 weeks, weights 280–300g) were provided by the Jining Lukang Experimental Animal Center, Jining, Shandong, China [Permit No. SCXK (LU) 20080002]. The protocol for this study was approved by the Faculty of Medicine and Health Sciences Ethics Committee for Animal Research, Affiliated Hospital of Shandong University of Traditional Chinese Medicine. Every effort was made to minimize any pain and distress experienced by the animals. Prior to initiating the experiments, the animals were housed for 1 week under conditions of ambient temperature (22 ± 2°C) and a 12:12 h light-dark cycle, with food and water available *ad libitum*.

### Chemical Sympathectomy

Twenty-four rats were randomly assigned to one of three groups: a normal control group (NC, n = 8), a sympathectomy control group (SC, n = 8) and a sympathectomy plus mecobalamin group (SM, n = 8). Rats in the NC and SC groups were given a single daily dose of 2 mL saline solution by gavage, while rats in the SM group received a single daily 2 mL dose of mecobalamin solution (0.15 mg/kg). The dosage of rats daily (mg / g) = human dosage daily (mg / 60kg) ×equivalent dosage coefficient / body weight of rat (g). The equivalent dosage coefficient of human to rat is 0.018 [18]. After continuous dosing for 7 days, all rats received an electrocardiogram and echocardiography, after which, sympathectomy was induced in the SC and SM rats by a single daily intraperitoneal injection of 6-OHDA (100 mg/kg; Sigma, St. Louis, Mo, USA, Cat. #162957) with a total volume of 2 mL/kg/d in 0.1% ascorbic acid/saline (Sigma, USA, Cat. # A8100), for three consecutive days[13]. Rats in the SM group continued to receive mecobalamin during their three days of 6-OHDA injections. Rats in the NC group were injected with the same volume of 0.1% ascorbic acid/saline.

### Electrocardiogram, echocardiography and mean arterial pressure

ECGs and echocardiography were performed prior to the first and after the last injection of 6-OHDA, under conditions of light anesthesia (sodium pentobarbital, 20 mg/kg, i.p.) as previously described [19]. All ECGs were recorded at a paper speed of 75 mm/sec, and the sensitivity was adjusted so that 1mV was equivalent to a 20mm deflections. Transthoracic echocardiography was performed by a professional technician using an M5 Vet Veterinary Ultrasound system (Mindray, Guangdong, China) to assess left ventricle (LV) function. Briefly, after shaving the chest, two-dimension (2D) short- and long- axis- directed M-mode and 2D recordings were acquired with a 15 MHz real-time microvisualization scan head probe. End systolic volume (ESV), end diastolic volume (EDV), stroke volume (SV), left ventricle ejection fraction (EF), cardiac output (CO) and LV stroke volume (SV) were recorded.

Blood pressure of rats was detected daily by the non-invasive rat tail method. Rats were put in the ALC-HTP animal system and heated to dilate rat tail artery, and data were measured with ALC-NIBP noninvasive blood pressure analysis system (ALCBIO, Shanghai, China) [20]. All rats were measured three times in parallel and mean arterial pressure (MAP) was recorded.

### Heart rate variability analysis

Heart rate variation (HRV) data were collected 24 hours per day during the three days of 6-OHDA injections. Readings of heart biopotential and physical activity were obtained using a biotelemetry system (Data Sciences Int., St Paul, MN, USA). A transmitter device was surgically implanted into the peritoneal cavity of each rat under general anaesthesia (sodium pentobarbital, 40 mg/kg, i.p.), and positioned with its electrodes in an Einthoven bipolar lead Ⅱ configuration (right foreleg and left hindleg). Following implantation, the rats were allowed to recover from the effects of surgery and anaesthesia for a period of 1 week, after which, the transmitters in the animals were activated using a magnetic switch. The sampling frequency for the electrocardiogram (ECG) was set to 1000 Hz, and ECG and physical activity data were collected to determine HRV. HRV data were analyzed using AcqKowledge 4.2 (Biopac Inc., Aero Camino, Goleta, CA, U.S.A.) and Kubios HRV 2.0 (University of Kuopio, Finland) software. The medium correction level in the Kubios software was selected to correct for artifacts. The power spectrum density estimation used in the frequency domain analysis of HRV was based on parametric Auto Regressive (AR) modeling methods [21].

The linear analysis of HRV included analyses of time and frequency domains. The main indexes of the time domain analysis were SDNN (the standard deviation of normal-to-normal RR intervals), SDANN (the standard deviation for the average value of each 5-min RR interval in 24 h), and RMSSD (the square root of the mean of the sum of the squares of differences between adjacent NN intervals). The main indexes of frequency domain analysis were low frequency (LF) (0.195–0.74 Hz), high frequency (HF) (0.74–2.5 Hz), total power(TP)(0–2.5Hz) and LF/HF. SDNN reflect the overall capacity for making heart rate adjustments [22]; RMSSD and HF reflect parasympathetic tone [23]; SDANN and LF are simultaneously affected by both sympathetic and parasympathetic nerves, but mainly reflect the sympathetic tone[24]; LF/HF reflects the balance between sympathetic and parasympathetic inputs to the heart [25].

### Collection of left ventricle tissue and myocardial histomorphology

Following 10 days of intragastric dosing, each rat was anesthetized with sodium pentobarbital (40 mg/kg, i.p.) and the transmitter device was removed. The LV of each rat was rapidly harvested and divided into three parts. One part was frozen in liquid nitrogen and serially sectioned at a thickness of 4 μm for HE staining, Masson staining and immunofluorescence assays, another part was fixed in 2.5% glutaraldehyde solution, and then observed under transmission electron microscope (TEM), while the last part was used for ELISA. Myocardial tissue for ELISA was homogenized in 10 volumes of buffer containing 10 mM Tris, 150 mM NaCl, and 1% Triton X-100, pH 7.4.

### Masson trichrome-stained sections

Collagen deposition in the LV was determined by Masson trichrome-staining Kit (Beijing Leagene Biotechnology Co.,Ltd,China, Cat. #DC0032). Collagen was stained blue in the paraffin tissue sections (4μm thick) according to the kit directions. Images were obtained in five random fields per section. Analysis was performed with ZEN Imaging analysis software (Carl Zeiss Jena, German). Area of blue staining was divided by the total area of microscopic fields.

### Sandwich enzyme-linked immunosorbent assay (ELISA)

The following molecules were purchased as standards to be used for ELISA determinations of their respective contents in LV tissue: tyrosine hydroxylase (TH) (Corporate R&D; Packaging Cat. # 10234R, Lot # 20120908), choline acetyltransferase (ChAT) (Corporate R&D; Packaging Cat. # 10538RT, Lot # 20120908), norepinephrine (NE) (Corporate R&D; Packaging Cat. # 10391RT, Lot # 20120908), nerve growth factor (NGF) (Abcam; Lot # ab100757, Hong Kong), interleukin-1β (IL-1β) (Abcam; Cat. # ab100767, Lot # GR127340-1), interleukin-6 (IL-6) (Abcam; Cat. # ab100772, Lot # GR12814-1), and tumor necrosis factor (TNF-α) (Abcam; Cat. # ab46070, Lot # GR126667-2). All assays were performed according to the manufacturer's instructions. All assay wells were developed with tetramethylbenzidine and measured at 450 nm. The protein content of each well was quantified using a standard curve constructed with known amounts of protein. All samples (1:10 dilution) were assayed in triplicate, and measurements are expressed as the mean value.

### Immunofluorescence staining of TH and ChAT in the LV

Tissue sections were immunostained at room temperature using standard procedures [26]. Noradrenergic nerve fibers were stained using mouse anti-rat TH (Millipore, MAB318, 1:1000 dilution, Billerica, MA, USA) and cholinergic cell bodies and nerve fibers were stained using rabbit anti-rat ChAT (Millipore, AB1582, 1:1000 dilution, Billerica, MA, USA). And then sections were incubated with FITC-conjugated AffiniPure goat anti-mouse IgG (ZSGB-Bio, ZF-0311, 1:100, Beijing, China) or TRITC-conjugated AffiniPure goat anti- rabbit IgG (ZSGB-Bio, ZF-0316,1:100, Beijing, China) at room temperature for 60 min. Tissue sections were then counterstained with 4',6-diamidino-2-phenylindole (DAPI, Solarbio, C0065, 0.5 mg/ml, Beijing, China) for 5 min at room temperature. Negative control sections processed without primary antibodies showed only background fluorescence. The stained slides were observed under Zeiss Vert A1 fluorescence microscope (Carl Zeiss Jena, German).

### Assessment of serum myocardial enzyme levels

Before and after injection of 6-OHDA, the tail of each rat was cut to enable collection of 1.5 mL blood. Levels of aspartate transaminase (AST), creatine kinase (CK), creatine kinase isoenzyme-MB (CK-MB) were determined using an AU 5400 automated analyzer provided by Olympus (Tokyo, Japan).

### Statistical analysis

Statistical analyses were performed using IBM SPSS Statistics for Windows, Version 19.0. Armonk, NY: IBM Corp. The normality of data distribution was evaluated using the Shapiro-Wilk test. Due to their skewed distributions, the parameters for frequency-domain analysis of HRV (LF, HF, and TP) and content of inflammatory cytokines (IL-1β, IL-6, and TNF-α) were transformed by calculating their natural logarithm to approximate normal distributions [27]. Differences between groups were analyzed using one-way analysis of variance (ANOVA), followed by Dunnett’s test or the Student-Newman-Keuls test. Categorical variables were compared using Fisher’s exact test. Differences between groups were considered statistically significant when the two-sided P-value was < 0.05.

## Results

### General condition of animals

No animal died following an intraperitoneal injection of 6-OHDA; however, the sympathectomised animals demonstrated significantly reduced levels of activity, and some rats displayed gross hematuria (7/8 in the SC group, 4/8 in the SM group, 0/8 in the NC group) and shivering.

### Evaluation of cardiac autonomic nerve damage

**Fig 1:**
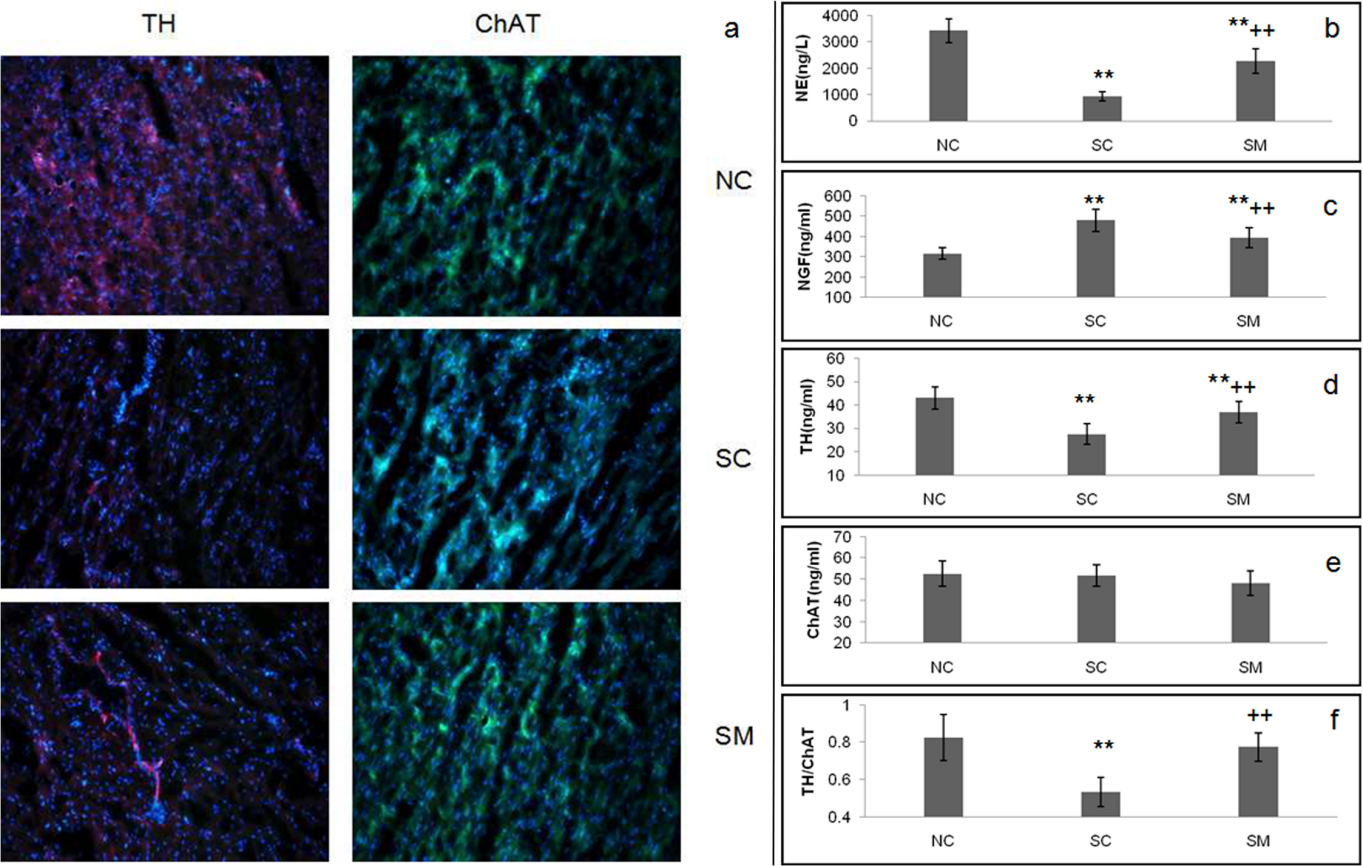
Characteristics of the sympathectomy model. Noradrenergic nerve fibers were labeled by FITC-conjugated anti-TH antibody (purple). Cholinergic nerve fibers were labeled by TRITC-conjugated choline anti-ChAT antibody (green) (bar 100um). Fig 1(b-f) are concentrations of NE, NGF, TH, ChAT in the LV homogenates by ELISA and the ratio of TH and ChAT. ** P<0.01 *vs*. NC group. ++P < 0.01 *vs*. SC group.

According to the immunofluorescence results, TH and ChAT are markers of sympathetic nerve activity and cholinergic neuron activity, respectively [28, 29]. In the NC group, nerve fibers positive for TH and ChAT were evenly distributed throughout the LV myocardium. In the SC group, the density of TH–positive nerve fibers was significantly reduced, whereas the density of ChAT-positive fibers was not changed. TH-positive expression was higher among rats in the SM group compared to rats in the SC group, but lower than rats in the NC group. There was no difference in density of ChAT-positive expression among the three groups (Fig 1A). These results demonstrated that 6-OHDA selectively destroyed sympathetic nerves, while leaving parasympathetic nerves intact.

According to the ELISA results, rats injected with 6-OHDA demonstrated 72.7% (P < 0.01) and 35.9% (P < 0.01) decreased concentrations of NE and TH, respectively in their myocardium homogenates; however, their concentrations of NGF increased by 52.1% (P < 0.01). Thus, rats in the SC group showed a chemical sympathetic denervation response similar to that previously described in the literatures [30, 31]. The degrees of decline in concentrations of NE and TH for rats in the SM group (33.6% and 13.9%, respectively; P < 0.01) were smaller than for rats in the SC. Additionally, NGF concentrations in the SM group were lower than those in the SC group. All three groups of rats (NC, SC and SM) showed similar concentrations of ChAT. The decrease in TH concentrations and unchanged ChAT concentrations resulted in the TH/ChAT ratio being reduced by 35.4% in the SC group and 6.1% in the SM group (Fig 1B–1F).

### Changes in HRV

**Fig 2:**
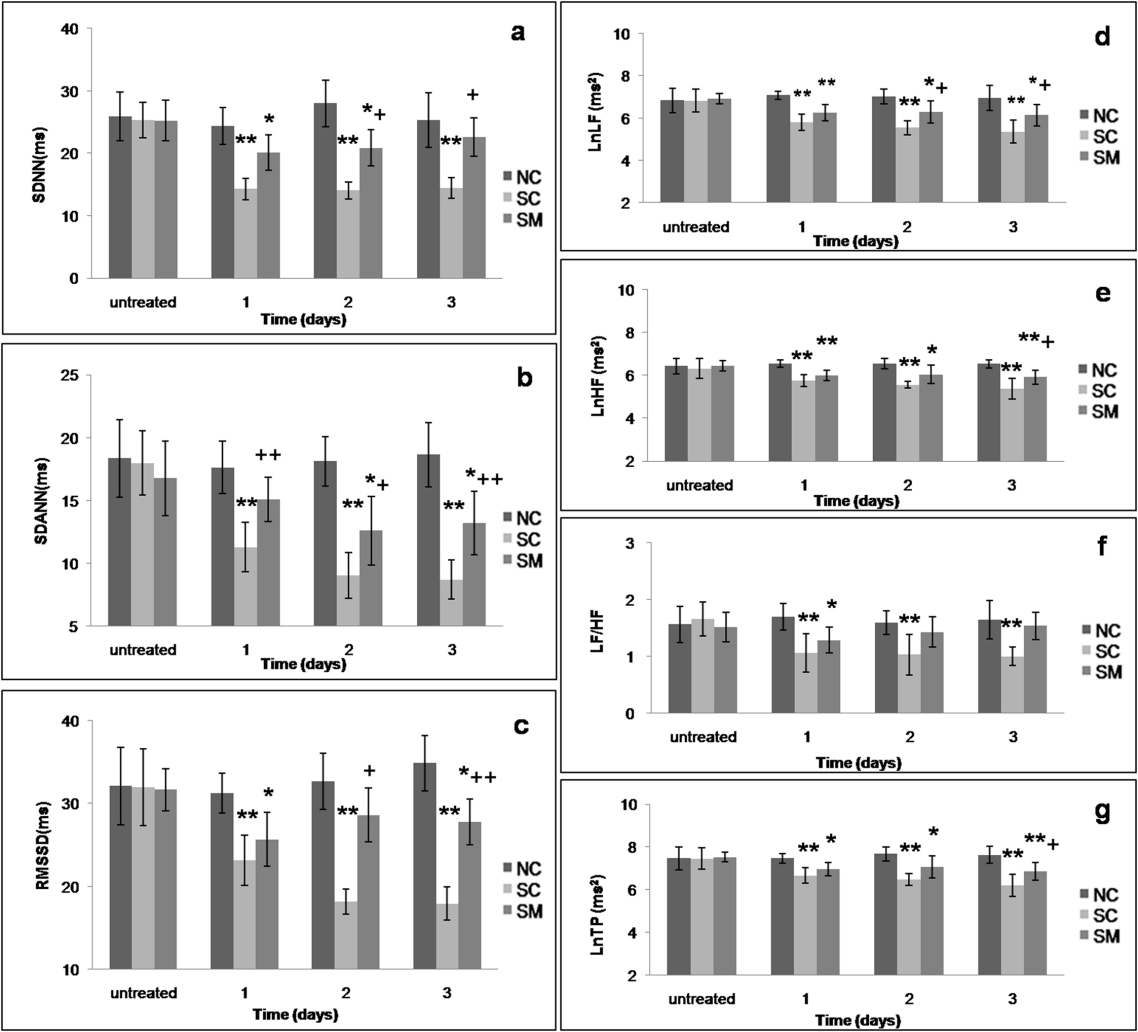
HRV analysis of rats. a-c: Dynamic changes in the time-domain analysis of HRV during three days of 6-OHDA injections. d-g. Dynamic changes in the frequency-domain analysis of HRV during three days of 6-OHDA injections. The parameters of frequency-domain analysis for HRV (LF, HF, and TP) were transformed by calculating their natural logarithms.* P<0.05,** P<0.01 *vs*. NC group. +P<0.05, ++P<0.01 *vs*. SC group.

An analysis of dynamic changes showed sharp declines in the HRV time-domain parameters SDNN, SDANN, and RMSSD (Fig 2A–2C) and frequency-domain parameters LF, HF,LF/HF and TP (Fig 2D–2G) during the 3 days of sympathectomy induction with 6-OHDA. All HRV parameters were significantly decreased at the first 2 days (P < 0.01, P < 0.05, *vs*. NC group and P < 0.01, P < 0.05, *vs*. SC group), while SDNN and LF/HF returned to normal levels on the third day in the SM group, suggesting the neuroprotective effect of mecobalamin.

### Changes in hemodynamic responses and electrocardiogram

**Fig 3:**
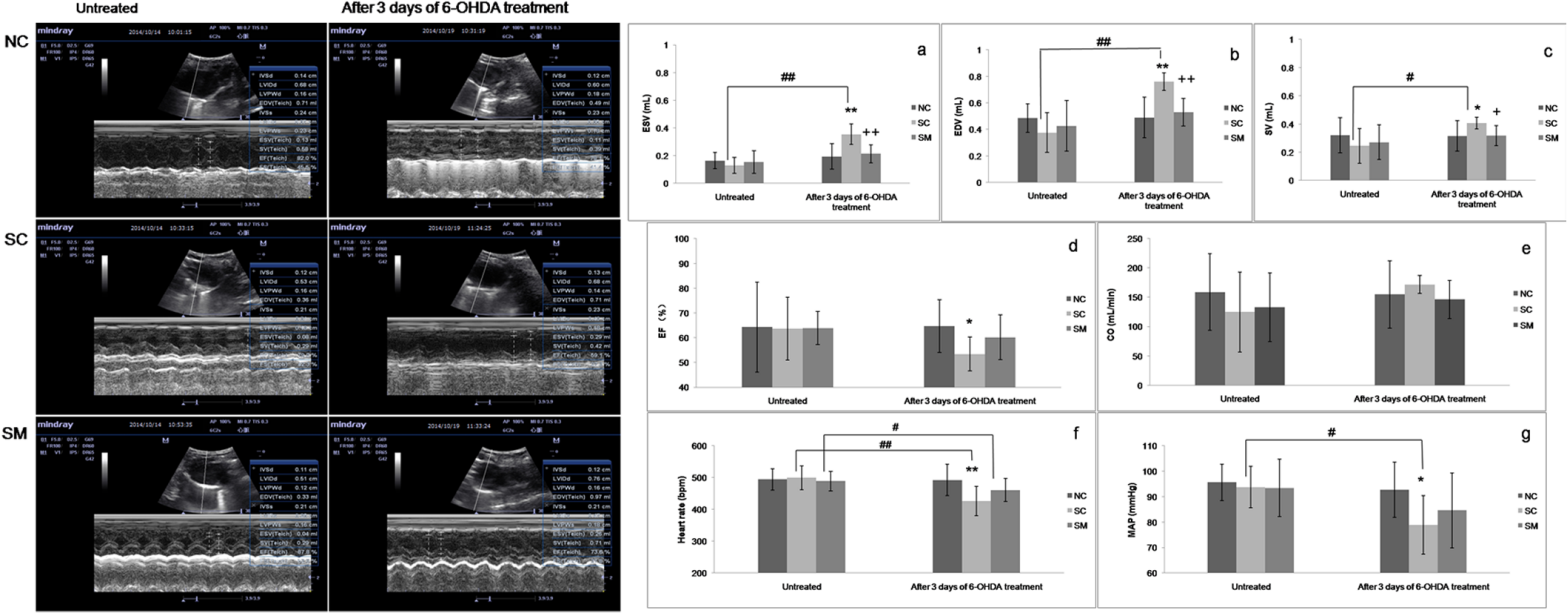
Changes in electrocardiogram and hemodynamic responses. * P < 0.05, ** P < 0.01 *vs*. NC group. +P < 0.05, ++P < 0.01 *vs*. SC group. #P < 0.05,## P < 0.01 *vs*. untreated value.

Echocardiographic was performed to estimate the cardiac function, representative images of the echocardiography were shown in the Fig 3. After injection of 6-OHDA, the HR (P<0.01) and MAP (P<0.05) declined significantly (Fig 3F and 3G). After 3 days of 6-OHDA treatment, rats in the SC group had higher levels of ESV, EDV and SV compared to values obtained pre-sympathectomy. When compared with rats in the NC group, the values of ESV (P<0.01), EDV (P<0.01) and SV (P<0.05) increased (Fig 3A–3C) while EF decreased (P<0.05) (Fig 3D).Rats in the SM group had lower value of ESV (P<0.01), EDV (P<0.01) and SV (P<0.05) than the SC group. Although CO increased slightly in SC group, there had no statistical significance among the three groups (Fig 3E).

**Fig 4:**
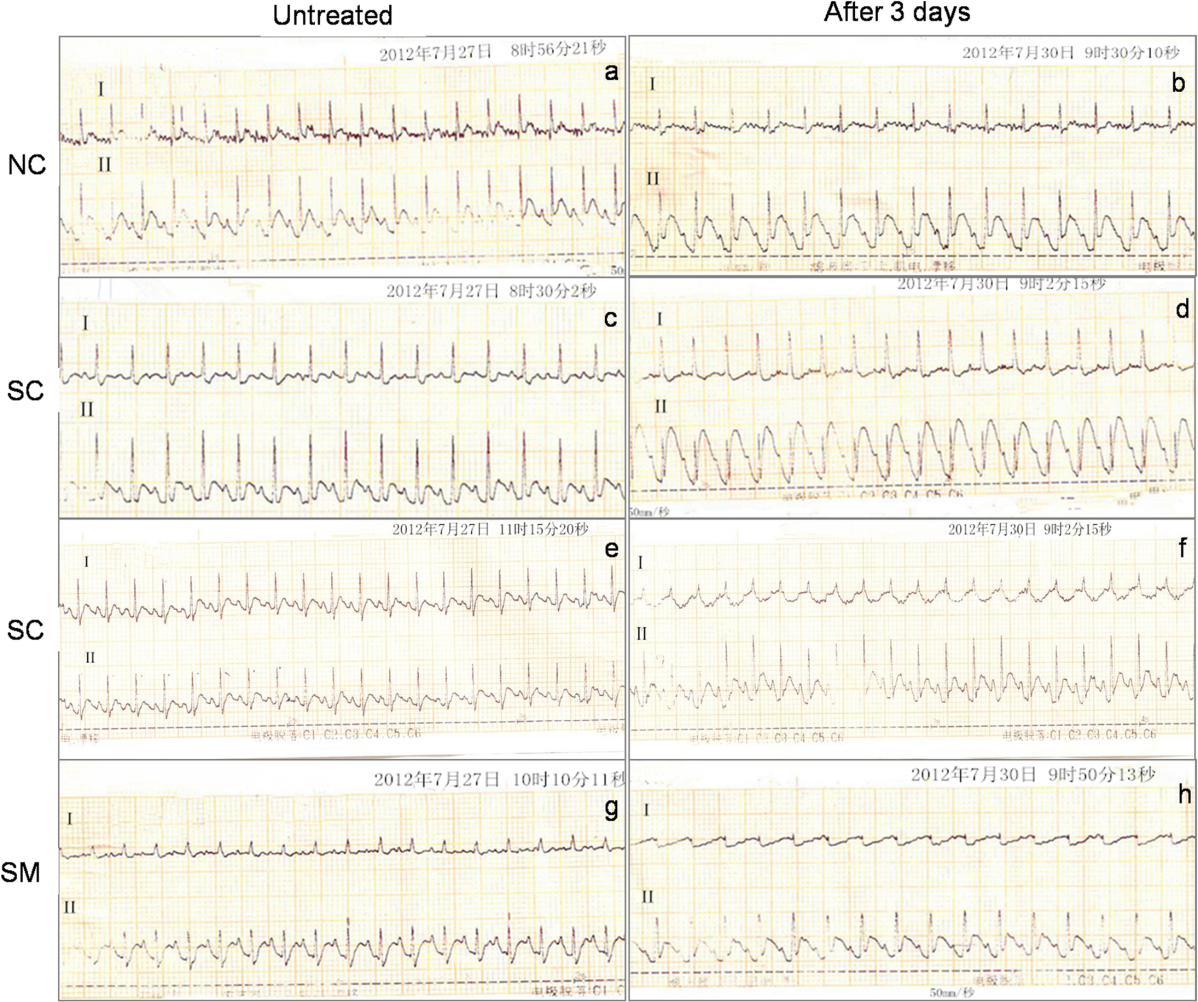
ECG changes in rats before and after treatment with 6-OHDA. There were no ECG changes among rats in the NC group (a, b). Rats in the SC group showed an elevated T-wave in lead Ⅱ (c, d) and an inverted T-wave in lead Ⅰ (e, f). Rats in the SM group exhibited an inverted T-wave in lead Ⅰ (g, h). ECGs were recorded with a paper speed of 75 mm/sec, and the sensitivity was adjusted so that 1mV was equivalent to a 20mm deflection.

Due to their good resolution of QRS components [32], we selected leads I and II to observe changes in ECG results after chemical sympathectomy. Abnormal T-wave changes were used as markers for myocardial injury [33]. Rats in the NC group showed no abnormal ECG changes after 3 days of dosing with saline solution (Fig 4A and 4B). However, elevated T-waves and inverted T-waves emerged in leads I and II among all rats in the SC group (Fig 4C–4F and Table 1). While ECG results in the SM group were better compared to those in the SC group, the difference was not statistically significant due to the small sample size (Fig 4G and 4H and Table 1).

**Table 1:** ECG changes by group (*n = 8* animals per group).

Group	Normal ECG	Abnormal ECG	Proportional number
**NC**	7	1	16.7%
**SC**	0	8	100%**
**SM**	3	5	83.3%*

Compared with the NC group

*P < 0.05

**P < 0.01.

### Release of myocardial enzymes

**Fig 5:**
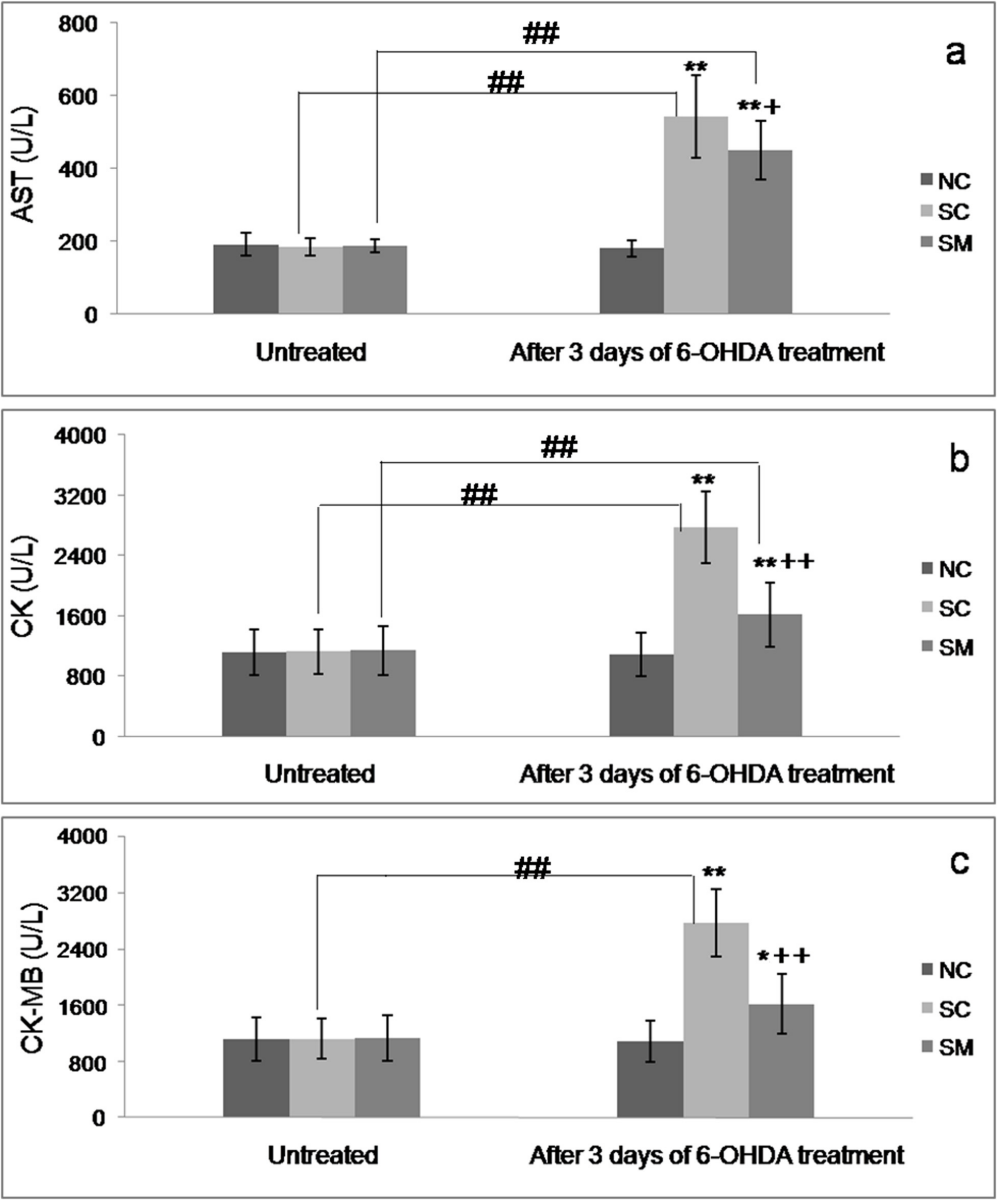
Changes in serum levels of cardiac biomarkers. * P < 0.05,** P < 0.01 *vs*. NC group. +P < 0.05, ++P < 0.01 *vs*. SC group. #P < 0.05, ## P < 0.01 *vs*. untreated value.

As shown in Fig 5, after 3 days of 6-OHDA treatment, rats in both the SC group and SM group had higher serum levels of AST (SC group: 542.5 U/L; SM group: 450.25 U/L) and CK (SC group: 2772.13 U/L; SM group: 1614.75 U/L) than before sympathectomy, and compared to rats in the NC group (P < 0.01). Only rats in the SC group had increased CK-MB levels (mean values increased from 1122.75 U/L to 2772.16 U/L) compared to their levels before treatment (P < 0.01). Compared with rats in the SC group, rats treated with mecobalamin had lower levels of AST, CK, and CK-MB (P< 0.05, P< 0.01).

### Myocardial morphology

**Fig 6:**
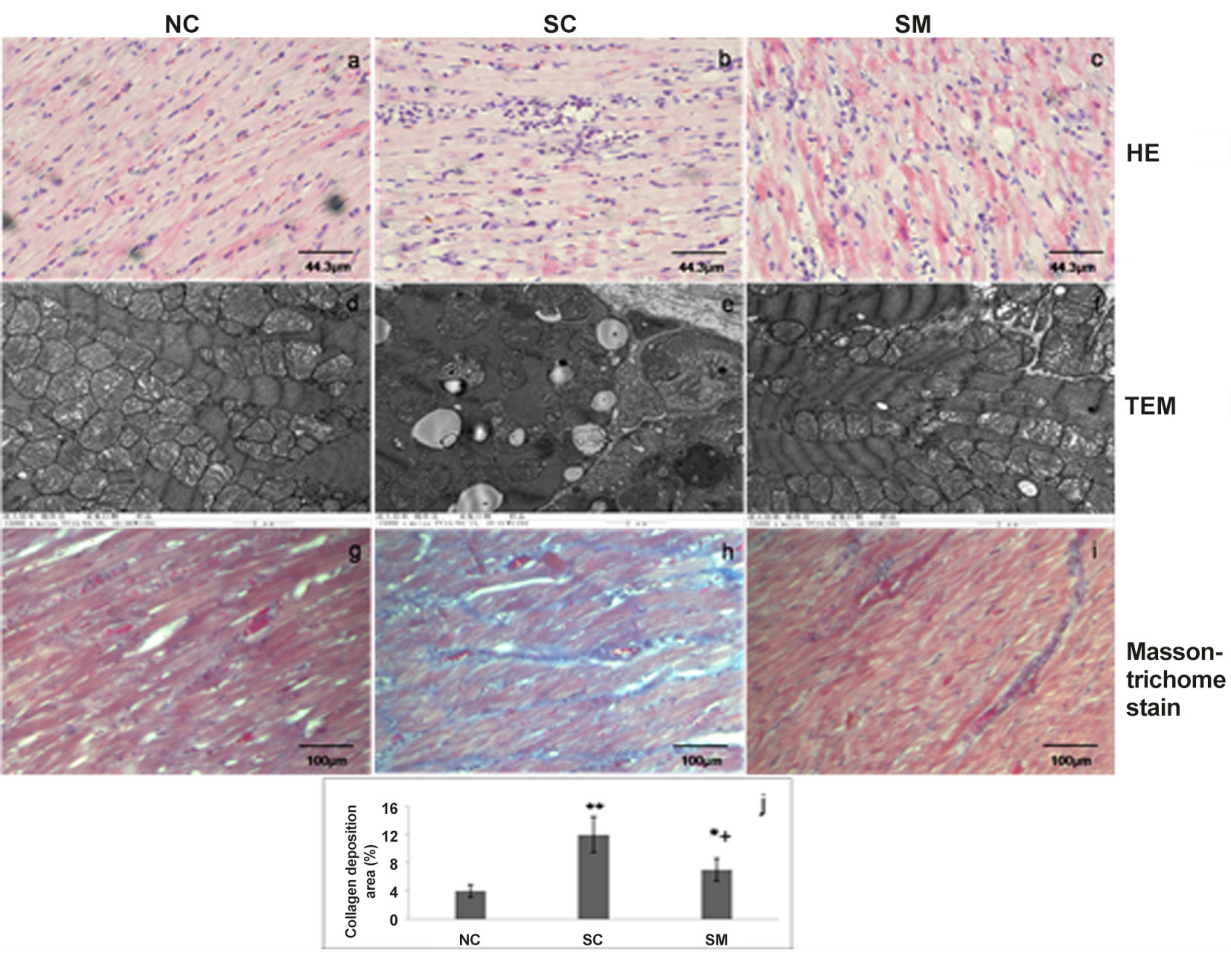
Myocardial morphology of the left ventricle after 6-OHDA injection. a-c. Photomicrographs of histological hematoxylin-eosin (HE). d-f. Transmission electron microscopic images of myocardial tissue. g-i. Collagen deposition in left ventricle examined by Masson-trichrome staining (blue staining). j. collagen depositon was presented by % collagen deposition to total area of the microscopic field. Data are expressed as means±SE (n = 8 animals per group). *P< 0.05, **P< 0.05 *vs*.NC group; +*P*<0.05 *vs*. SC group.

In the normal rats, myocardial cells were neatly arranged, sarcomeres were arranged regularly, subcellular structure, such as mitochondrias, were rich, clustered and in normal morphology (Fig 6A and 6D). However, 6-OHDA injections induced cardiomyocytes degeneration and necrosis and focal inflammatory cell infiltration as well as interstitial connective tissues hyperplasia. Sarcomere disorder, myofilament dissolved and fatty degeneration, mitochondria swelling, cristae vacuolation was found in myocardial submorphology of SC group by TEM (Fig 6B and 6E). Additionally, increased collagen deposition in myocardial tissues from rats in the SC group was examined by Masson-trichome staining (blue staining) (Fig 6H). Myocardial tissues from the SM group showed trends of reduced inflammatory cell infiltration and improved myocardial tissues morphology compared to tissues obtained from the SC group (Fig 6C, 6F and 6I). As mecobalamin could prevent myocardial injury in some degree, suggesting that the disordered structure was, at least partly, attributable to effect of disruption of sympathetic nerves on vasculature and myocytes.

### Concentrations of inflammatory cytokines in the LV

**Fig 7:**
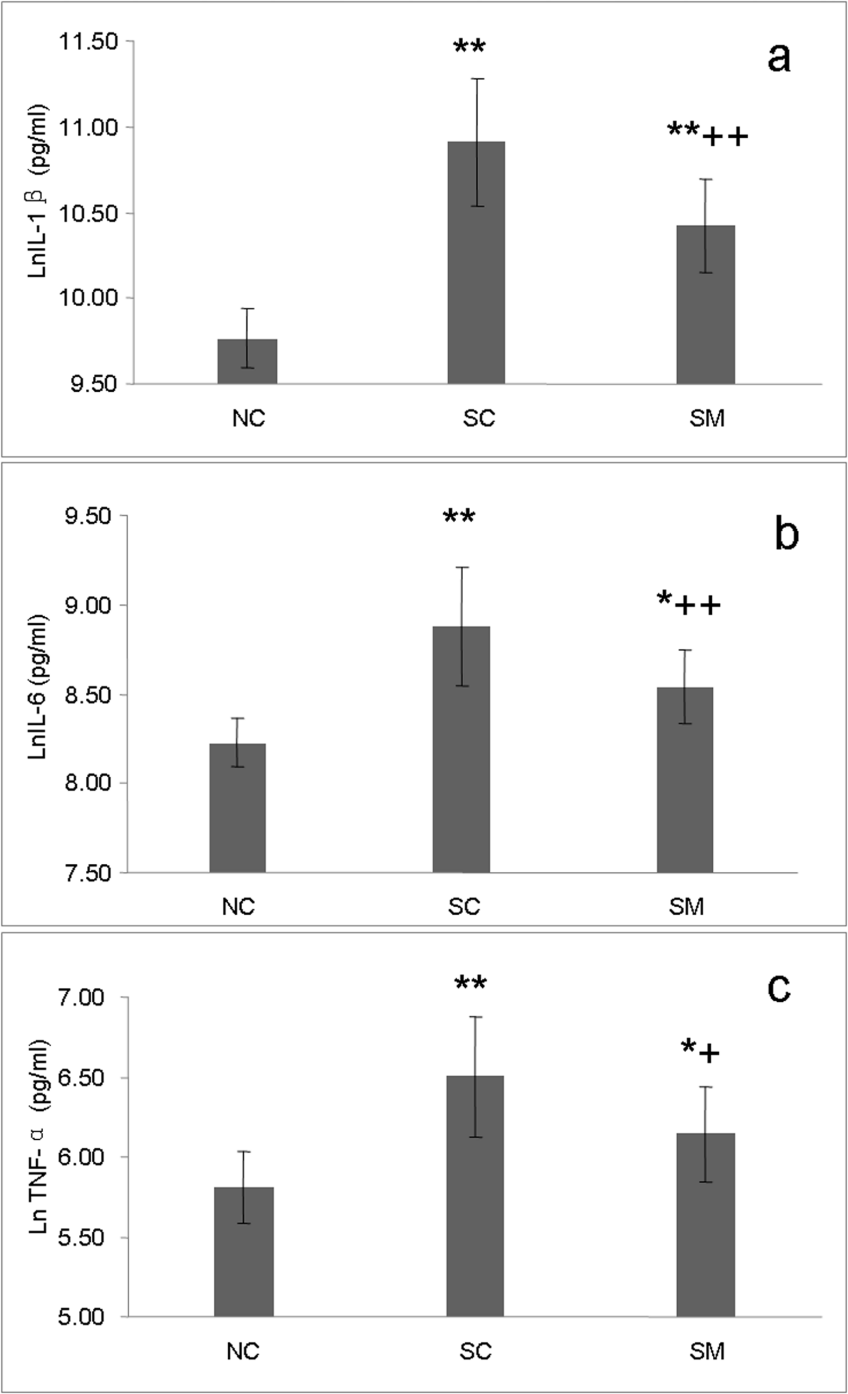
Concentrations of inflammatory cytokines in the LV. Concentrations of IL-1β, IL-6 and TNF-α in LV homogenates by ELISA. The concentrations of IL-1β, IL-6 and TNF-α were transformed by calculating their natural logarithms.* P<0.05, **P<0.01, *vs*. NC group. +P<0.05, ++P<0.01, *vs*. SC group.

Following 6-OHDA treatment, concentrations of inflammatory cytokines in myocardial homogenates increased significantly (Fig 7). In the SC group, the concentrations of IL-1β, IL-6, and TNF-α increased by 237%,126%, and 106%, respectively, and peaked at levels of 66502.23 (35655.25–74699.92) pg/mL, 1591.99 (989.83–1956.32) pg/mL, and 758.93 (469.23–874.16) pg/mL, respectively. Although inflammatory cytokines in the SM group also showed an upward trend, they remained much lower compared to levels in the SC group, with IL-1β, IL-6, and TNF-α decreasing by 41.6%, 30.8%, and 29.7%, respectively.

## Discussion

The major results of the present study are as follows: (1) Following induction of sympathectomy in rats with 6-OHDA, HRV was reduced, and parameters inferring parasympathetic activity (RMSSD and HF) were significantly decreased. (2) LV contractile dysfunction, abnormal ECG, hemodynamic and myocardial morphology changes as well as elevated levels of myocyte injury biomarkers were observed. (3) Excessive expression of inflammatory cytokines was observed following injections of 6-OHDA. However, mecobalamin reduced 6-OHDA-induced sympathetic damage and myocardial injury.

In the present study, we showed that chemical sympathectomy with 6-OHDA led to cardiac sympathetic neurodegeneration in rats, which agreed with previous studies that showed significant reductions in global cardiac TH expression and catecholamine levels, and increased NGF levels as a result of exposure to 6-OHDA [11, 34].

HRV can quantitatively reflect the activity levels of sympathetic and parasympathetic nerves. Following induction of sympathectomy, values for SDNN and TP, which represent autonomic regulation of cardiac function, were decreased, and the reduced LF peaks and LF/HF ratios indicated that the sympathetic-vagal balance in the heart had been replaced by parasympathetic dominance. Additionally, SDANN, which is the most controversial parameter of HRV, was also reduced. Most previous studies either found that SDANN was influenced by both sympathetic and parasympathetic activities [35], or regarded SDANN as an index of sympathetic tone [22]. Zulfiqar et al [22]proposed that SDANN reflects HRV-sympathetic function, and continues to decrease throughout life. Other studies conducted in healthy subjects or patients with heart failure, reported that SDANN was higher during activity than during rest, decreased at night-time (the period of sympathetic inactivity with elevated vagal modulation), and then increased during the daytime (the period with higher sympathetic modulation) [36–38]. Thus, we preferred to regard SDANN as an indicator of sympathetic nerve activity. In our study, injection of 6-OHDA destroyed sympathetic nerve activity, and caused SDANN declined significantly. Although SDANN values showed a slight decline in rats treated with mecobalamin (SM group), the declines were less than those in the SC group, suggesting that mecobalamin may protect against sympathetic destruction caused by 6-OHDA. It is generally believed that 6-OHDA selectively destroys sympathetic nerves, and has no impact on parasympathetic nerves. However, we observed that the HRV parameters RMSSD and HF, which are mainly determined by parasympathetic activity, were reduced in 6-OHDA treated rats. Conversely, distribution of the parasympathetic marker ChAT in the LV was unchanged. Most previous reports have emphasized the opposition between sympathetic and parasympathetic nervous systems, and neglected their synergistic functions. Koizu et al [39] reported that simultaneous stimulation of both sympathetic and vagal nerves produced a greater increase in CO compared to that resulting from isolated stimulation of either nerve alone. Herring’s studies [40, 41] described the anatomical basis for the sympathovagal cross talk model, neuropeptide Y and galanin released from sympathetic nerve inhibited the vagal tone via a reduction in acetylcholine release. Both the above studies support the theory that the two branches of the autonomic nervous system interact with each other, and vagal nerves dysfunction can result from destruction of sympathetic nerves, which may weaken sympathetic-vagal synergistic actions, resulting in diminished regulation of vagal functions. Therefore, maintaining an intact cardiac sympathetic system and improving sympathetic denervation should benefit functions of vagal nerves and sympathetic-vagal synergistic actions, and assist in regaining normal regulation of the cardiac autonomic nervous system as well as sympathetic-vagal balance.

Many animal studies have demonstrated the effects of cardiac sympathetic denervation on cardiac function and structure in the pathological conditions; however, there few study illustrated the effect in normal condition. In the present study, we observed cardiac function and structure changes during the process of the chemical sympathetomy with 6-OHDA. After sympathectomy induced by 6-OHDA, rats in the SC group showed increased EDV and SV, which may be attribute to the enhanced filling time caused by the reduced HR. The preserved CO and decreased MAP indicated that total peripheral vascular resistance must be decreased after chemical sympathectomy with 6-OHDA in the SC group, and the reduced peripheral vascular resistance decreases the afterload may also contribute to the increased SV. The increased ESV and diminished EF values reflected the LV myocardial contractility dysfunction. We performed whole body sympathectomy by intraperitoneal injection of 6-OHDA, this may disrupt all sympathetic nerve including the renal sympathetic nerve. However, previous experiments demonstrated that in normal rats, renal denervation did not significantly alter or slightly reduced the arterial pressure, HR and renal hemodynamics [42–45]. Therefore, we deduced the hemodynamic changes presented in our study cannot be attributed to the knock-effect renal sympathetic denervation.

Rats in our study were evaluated by an ECG both before and after treatment with 6-OHDA. Two main factors might account for the abnormal T-wave changes seen after injection of 6-OHDA. Firstly, such changes might have been a direct affect of sympathetic denervation. It was previously reported that changes in sympathetic tone can produce neurogenic changes in electrocardiographic results [10, 46]. The second factor is that a myocardial injury may have occurred during or after chemical sympathectomy. Thus, cardiac denervation which occurred in the absence of a prior myocardial injury in the present study might have facilitated abnormal ECG changes. Biochemical markers CK, CK-MB and AST have been used for the detection of heart damage in experimental animal [47]. CK-MB and CK were released from the myocardium into the plasma with progression of cardiomyocytes damages and considered as an important marker of myocyte injury [48]. Although AST is a non-specific heart lesion, increase in its serum activity is detected when there is severe myocardium damage. Moreover, AST serum measurement is frequently associated with CK dosage, a marker to complement changes observed in CK serum activity [49]. The elevated serum levels of CK, CK-MB and AST, and observed microstructural histopathological changes were consistent with abnormal ECG changes, and suggested that cardiac tissue was impaired after injection of 6-OHDA. Facoetti et al [50] reported that abrogation of cardiac sympathetic efferent neurons with 6-OHDA caused significant mast cell activation which was known to stimulate fibroblast growth and collagen synthesis, the enhanced mast cell activation caused by sympathetic denervation was likely responsible for the interstitial collagen deposition found in cardiac tissue. This may partly explain the increased collagen deposition in chemical sympathectomised rats having undergone in our study.

Conversely, Brum P et al [51] found that chronic elevation of sympathetic tone in the absence of prior abnormal alternations in myocardial structure or function can lead to direct myocardial injury. Therefore, combined with our study, it suggested that both cardiac sympathetic hyperinnervation and denervation can induce alterations in normal myocardial tissue structure, and cause myocardial injury. Thus, the capacity to maintain sympathetic nerves within a certain physiological range is important for ensuring proper cardiac function and tissue structure.

In order to exclude the possibility that the myocardial injuries found in rats resulted from the intrinsic cardiotoxicity of 6-OHDA [52]. Mecobalamin was used to alleviate damage to sympathetic nerves in the SM group. Previous studies have suggested the efficacy of mecobalamin for improving diabetes-related cardiovascular autonomic disorders [53]. Mecobalamin has been shown to act on downstream mechanisms involving NGF and brain-derived neurotrophic factor to promote neurite outgrowth and the regeneration and conduction of nerves [15]. Additionally, mecobalamin displays a special affinity for nerve tissue, and promotes myelination and transport of the axonal cytoskeleton [54]. In our current study, according to the changed HRV parameters and contents of NE, NGF and TH in the cardiac tissue, rats administrated with mecobalamin have a slighter nerve injury compared with rats without mecobalamin. It suggested that rats pre-treated with mecobalamin displayed lesser degrees of cardiac sympathetic denervation compared with rats in the SC group. These results indicated that mecobalamin can protect against cardiac sympathetic nerve destruction caused by 6-OHDA. Meanwhile, compared with rats in the SC group, rats pre-treated with mecobalamin showed better ECG results, lower cardiac enzyme levels, and more favorable histopathologic changes in their cardiac tissue, suggesting that their lesser degrees of cardiac sympathetic denervation were related to these more favorable findings. Therefore, we believe that the lesser degrees of myocardial injury found in the SM group must be partially attributed to the protective effect of mecobalamin on the cardiac sympathetic nervous system.

A previous study reported that rats treated with 6-OHDA showed evidence of lymphocyte and neutrophil infiltration into their myocardial tissue and surrounding coronary vessels [11]. In our study, we also observed infiltration of inflammatory cells into the cardiac tissue of rats in the SC group. Excessively high levels of inflammatory cytokines such as IL-1β, IL-6, and TNF-α are known to cause or aggravate myocardial injury and promote cardiac remodeling [55, 56]. Substantial evidence suggests that the autonomic nervous system communicates with the immune system to modulate inflammatory reactions [57, 58]. The sympathetic nervous system plays a dual role in regulating inflammation, because it mediates both pro- and anti-inflammatory activities [57]. It has been suggested that by stimulating the β2-adrenoreceptor-cAMP-protein kinase A pathway, NE inhibit the production of pro-inflammatory cytokines and stimulate the production of anti-inflammatory cytokines [59]. Our study results showed elevated levels of pro-inflammatory cytokines (IL-1β, IL-6, and TNF-α) in the myocardial tissue of rats having undergone sympathectomy, whereas rats treated with mecobalamin shown milder sympathetic destruction and lower relative levels of inflammatory factors. This result appears to support the above-mentioned views, and suggests that chemical sympathectomy may partially contribute to overexpression of pro-inflammation cytokines. This may be one of the potential mechanisms that explaining the myocardial injury after chemical sympathectomy induced by 6-OHDA.

### Study Limitations

Our current study has several limitations that should be mentioned. First, various complex pathological factors contribute to heart disorders in humans, and many of these factors may not be adequately modeled by the cardiac sympathectomy induced by injection of 6-OHDA. However, 6-OHDA mainly acts by destroying the nerve terminal which is different with the surgical sympathectomy, and therefore produces damage similar to sympathetic damage caused by degradation of synaptic structures in humans [7]. Second, chemical sympathectomy with 6-OHDA is one of the most used methods of sympathetic degeneration, however, its intrinsic toxicity must be considered and excluded. Additionally, intraperitoneal injection of 6-OHDA causes whole body sympathetic nerve denervation, including the peripheral vascular and influence the hemodynamic. Therefore, our further study needs to found a better method which creates direct denervation of the cardiac sympathetic.

## Conclusions

Our study showed that 6-OHDA treatment resulted in dysregulation of the cardiac autonomic nervous system. Myocardial injuries and inflammatory reactions in the heart after 6-OHDA treatment can be partially attributed to cardiac sympathetic denervation. Mecobalamin protected cardiac sympathetic nerves, and subsequently alleviated myocardial injuries and inflammatory reactions.
